# Preparation and Failure Behavior of Gel Electrolytes for Multilayer Structure Lithium Metal Solid-State Batteries

**DOI:** 10.3390/gels11080573

**Published:** 2025-07-23

**Authors:** Chu Chen, Wendong Qin, Qiankun Hun, Yujiang Wang, Xinghua Liang, Renji Tan, Junming Li, Yifeng Guo

**Affiliations:** 1 Guangxi Key Laboratory of Automobile Components and Vehicle Technology, Guangxi University of Science & Technology, Liuzhou 545006, China; 13434119278@163.com (C.C.); qinwendong@163.com (W.Q.); 13383371921@163.com (Q.H.); 18068533778@163.com (R.T.); jmli@gxust.edu.cn (J.L.); 2 School of Mechanical Engineering, Chengdu University, Chengdu 610106, China; guobujia2000@163.com

**Keywords:** gel polymer electrolyte, multilayer structure, lithium metal, performance and failure behavior, solid-state batteries

## Abstract

High safety gel polymer electrolyte (GPE) is used in lithium metal solid state batteries, which has the advantages of high energy density, wide temperature range, high safety, and is considered as a subversive new generation battery technology. However, solid-state lithium batteries with multiple layers and large capacity currently have poor cycle life and a large gap between the actual output cycle capacity retention rate and the theoretical level. In this paper, polyvinylidene fluoride-hexafluoropropylene (PVDF-HFP)/polyacrylonitrile (PAN)—lithium perchlorate (LiClO_4_)—lithium lanthanum zirconium tantalate (LLZTO) gel polymer electrolytes was prepared by UV curing process using a UV curing machine at a speed of 0.01 m/min for 10 s, with the temperature controlled at 30 °C and wavelength 365 nm. In order to study the performance and failure mechanism of multilayer solid state batteries, single and three layers of solid state batteries with ceramic/polymer composite gel electrolyte were assembled. The results show that the rate and cycle performance of single-layer solid state battery with gel electrolyte are better than those of three-layer solid state battery. As the number of cycles increases, the interface impedance of both single-layer and three-layer electrolyte membrane solid-state batteries shows an increasing trend. Specifically, the three-layer battery impedance increased from 17 Ω to 42 Ω after 100 cycles, while the single-layer battery showed a smaller increase, from 2.2 Ω to 4.8 Ω, indicating better interfacial stability. After 100 cycles, the interface impedance of multi-layer solid-state batteries increases by 9.61 times that of single-layer batteries. After 100 cycles, the corresponding capacity retention rates were 48.9% and 15.6%, respectively. This work provides a new strategy for large capacity solid state batteries with gel electrolyte design.

## 1. Introduction

With the rapid development of electronic technology, the energy density of traditional batteries can no longer meet the modern demand for high-energy batteries. Currently, the development of batteries faces two main challenges: how to improve the energy density of batteries and how to enhance battery safety. Compared with traditional liquid lithium-ion batteries, the most remarkable feature of solid-state batteries is the use of solid electrolytes to replace flammable liquid electrolytes. In terms of safety, when liquid batteries encounter overcharging, overheating, or external impacts, the flammable liquid electrolytes are extremely prone to causing fires or even explosions. Solid-state batteries, however, fundamentally avoid this risk, significantly improving operational safety. In terms of energy density, solid-state batteries perform even more impressively. Under the same volume or weight, they can store more electrical energy. Additionally, solid-state batteries have a longer cycle life, capable of withstanding more charge-discharge cycles, which effectively reduces battery performance degradation and lowers the cost and frequency of battery replacement. Precisely due to these outstanding advantages, solid-state batteries have become the focus of research and development for numerous scientific teams and enterprises [1,2,3,4,5,6]. Currently, polymer electrolytes [7,8,9,10,11,12,13], ceramics materials [14,15,16,17,18,19,20] and ceramic/polymer composite electrolytes [21,22,23,24,25,26] have been intensively studied. Although rubbery plastic electrolytes like that are easy to process, they are more than 60 microns in thickness, resulting in more useless space in the battery. Besides, used on the market. Ceramic materials have good electrical conductivity, but are brittle and hard, and they are prone to cracking when slightly bent. As for all-solid-state lithium batteries, although their safety has been improved, they still cannot beat the current liquid batteries in terms of fast charging speed and energy storage capabilities. There is a very interesting contradiction here. It seems that one of them must be sacrificed for safety and high performance. How to find the balance point has become the focus of research. However, the larger thickness of the gel polymer electrolyte (>60 μm) increases the volume proportion of inactive components in the battery, and its electrical conductivity is much worse than the liquid electrolytes. As for all-solid-state lithium batteries (ASSLB), although their safety has been improved, they still cannot beat the current liquid batteries in terms of fast charging speed and energy storage capabilities [27,28,29,30].

Gel polymer electrolyte membranes (GPEs) are similar to existing commercial separators in terms of thickness and cycle life, which lays the foundation for the rapid promotion of long-life solid-state batteries [31]. This type of material also has the characteristics of light weight and low processing equipment cost, especially its relatively simple preparation process and an electrochemical window that can be adjusted. These characteristics make it an ideal option for large-scale production of solid-state batteries [32,33]. Specifically, polyvinylidene fluoride (PVDF) materials have a promoting effect on lithium-ion transmission because they contain strong polar C-F bonds and high dielectric constants. The PVDF-HFP copolymer formed by the introduction of hexafluoropropylene can not only effectively dissolve the lithium salt, but also exhibit excellent chemical stability and film-forming characteristics. Polyacrylonitrile (PAN) exhibits unique advantages when comparing different polymer materials. This material has a low molecular orbital energy level without occupies a low molecular orbital energy level, has a wide electrochemical window and high voltage stability, and is especially suitable for lithium metal battery systems that match high voltage positive electrodes. What is more worthy of attention is that the C≡N group in the PAN molecular chain can form a coordination effect with Li^+^. This characteristic not only promotes the dissociation of lithium salts, but also effectively inhibits the growth of lithium dendrites in the composite gel electrolyte. It can be seen that the coordinated use of PVDF-HFP/PAN provides a new direction for the development of lithium metal battery electrolytes that combine high energy density and safety. Zhang et al. [34] dispersed lithium lanthanum zirconium tantalate (LLZTO) ceramic powder in a polymer matrix and successfully prepared PVDF/LLZTO composite electrolyte. In the LiCoO_2_/Li battery composed of this composite electrolyte, the initial specific discharge capacity reached 150 mAh·g^−1^. After 120 cycles at 0.4 C, the capacity retention rate of the battery can reach 98%. Gu et al. [35] PVDF-HFP/LLZTO composite GPEs with reported a simple synthesis route and readily available raw materials. The composite electrolyte exhibited a wide electrochemical stability window (about 5 V), with a specific capacity of about 150 mAh·g^−1^ at 0.1 C, and a coulombic efficiency of about 99% after 50 cycles. Professor Liang Feng’s team from Kunming University of Science and Technology has proposed a new strategy for the high-efficiency ultraviolet (UV) curing preparation of composite solid-state electrolytes with high ionic conductivity. This strategy enables the rapid preparation of composite solid-state electrolytes within 45 s. Through solvation structure regulation and in-situ curing, it simultaneously constructs a stable interfacial phase and a tight interface, effectively inhibiting battery dendrite growth and interfacial reactions, reducing battery interface impedance, and achieving long cycle life of the battery [36]. The UV curing technology provides a new idea for the commercialization of solid-state electrolyte membranes. However, it has relatively insufficient experience in industrial applications, requiring further verification of long-term stability and integration with other strategies (such as three-dimensional framework current collectors) to enhance practicality.

Multilayer pouch cells have gained widespread adoption in consumer electronics, electric vehicles, and energy storage systems, primarily owing to their high energy density, design flexibility, and relatively low weight [37]. However, they are inherently challenged by several critical issues. The utilization of aluminum laminate film as the casing material, while lightweight, results in significantly inferior mechanical strength compared to metallic (steel or aluminum) hard-case batteries. Furthermore, gas generation during cycling or under elevated temperatures leads to cell swelling (“pouch bulge”) [38,39]. The flexible pouch structure provides inadequate constraint against this expansion, and persistent swelling can progressively stretch and weaken the aluminum laminate film, thereby increasing the risk of rupture and potentially compromising the integrity of the enclosing module structure. Thermal management presents another significant challenge within the multilayer stacked configuration. Heat generated internally must dissipate through the stacked cell layers to the surface and then to the external environment, encountering relatively high thermal resistance. This impedes efficient heat dissipation from the core region, promoting the formation of localized hotspots [40,41]. The heat sealing process of the aluminum laminate film is critical for ensuring battery safety. The seal edges are susceptible to failure due to various factors, including suboptimal sealing parameters (e.g., improper temperature, pressure, or duration control), material defects, long-term electrolyte corrosion, and mechanical stresses (e.g., vibration, swelling-induced stress). Such seal integrity breaches pose substantial safety and reliability risks. While multilayer pouch cells offer distinct advantages in energy density and design versatility, the aforementioned challenges—particularly concerning safety, longevity, cost reduction, and manufacturability at scale—remain the focus of intensive research and engineering efforts aimed at enabling their broader application.

In this paper, PVDF-HFP/PAN-LiClO_4_-LLZTO composite gel electrolyte was prepared using a UV curing process, single-layer soft-pack batteries and three-layer soft-pack batteries were assembled, and the cycle life was compared and tested. The different reasons for the capacity attenuation of three-layer soft-pack batteries and single-layer soft-pack batteries were analyzed, and the capacity attenuation of multi-layer soft-pack batteries and single-layer soft-pack batteries was investigated. The electrochemical behavior of soft-pack batteries is studied to explore ways to improve the cycle life of multilayer soft-pack batteries in order to promote their industrialization.

## 2. Results and Discussion

**Figure 1:**
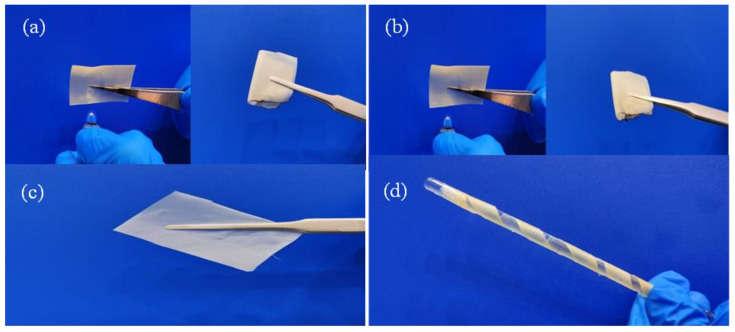
(**a**) GPEs ignited for 1 s; (**b**) GPEs ignited for 5 s; (**c**) GPEs tile; (**d**) After GPEs bending.

Figure 1a,b are the experimental pictures of the ignition of GPEs film for 1 s and 5 s respectively. It can be seen from the experiment that the film can not be ignited, indicating that the GPEs film is nonflammable. Figure 1c,d are the comparison diagrams of GPEs film before and after bending respectively. It can be seen from the diagram that the film has basically no change before and after bending, indicating that the film has good flexibility.

**Figure 2:**
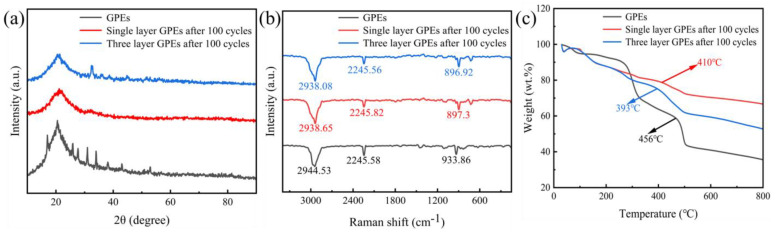
(**a**) XRD, (**b**) Raman, and (**c**) TG of GPEs.

Observing the phase analysis data in Figure 2 can reveal more important information. In the X-ray diffraction (XRD) pattern of Figure 2a, the PVDF-HFP matrix in the original state shows a typical crystal diffraction peak at 20.7° in 2θ, and the characteristic peak of the PAN component is located near 17° in 2θ. It is worth noting that in the three-layer soft-pack battery samples after 100 charge and discharge cycles, these characteristic peaks significantly weakened or even disappeared. This change in phase structure suggests that the ion transport channel inside the electrolyte may have been damaged and the stability of the gel system has dropped significantly. The Raman spectrum in Figure 2b further confirms this conclusion. The characteristic vibration peaks that should appear in the three-layer structure sample completely disappear in the single-layer sample, which is mutually verified with the XRD detection results. In addition, the thermogravimetric analysis curve of Figure 2c shows the decomposition behavior of the material at high temperatures, and its thermal stability characteristics also provide an important reference for subsequent research. The above experimental results generally show that although GPEs materials have excellent flame retardancy and mechanical properties in the initial state, their internal structure will undergo irreversible changes after long-term use. The stability of this material during recycling, especially the disintegration of the crystal structure and changes in chemical composition, may be key factors that restrict their practical application. Follow up research needs to conduct a more in depth discussion on these failure mechanisms.

Thermogravimetric analysis (TGA) results are shown in Figure 2c that in the temperature range of 30~800 °C, the weight loss temperature of GPEs before charge and discharge cycle is about 456 °C, while the weight loss temperature of single-layer and multi-layer GPEs after 100 charge and discharge cycles is about 400 °C. When the temperature reaches 800 °C, the weight loss of single-layer and multi-layer GPEs before charge and discharge cycle is 33.37% and 47.28%, respectively, indicating that the weight loss rate decreases after 100 charge and discharge cycles. The slope of the TGA curve becomes less steep after 100 cycles, which implies a slower decomposition or evaporation rate of the components under heating. This reduced weight loss rate suggests that the structure of the GPEs or the interface between GPEs and electrode has become more stable, likely due to the formation of a stable passivation layer or improved interfacial contact during cycling. After 100 charge and discharge cycles, the TGA curve shows a more gradual weight loss, indicating a reduced thermal decomposition rate, which may be attributed to the enhanced stability of the GPEs structure or the formation of a stable interfacial layer. Combined with the analysis of XRD and Raman spectroscopy results, it is shown that GPEs has decomposed at this time.

**Figure 3:**
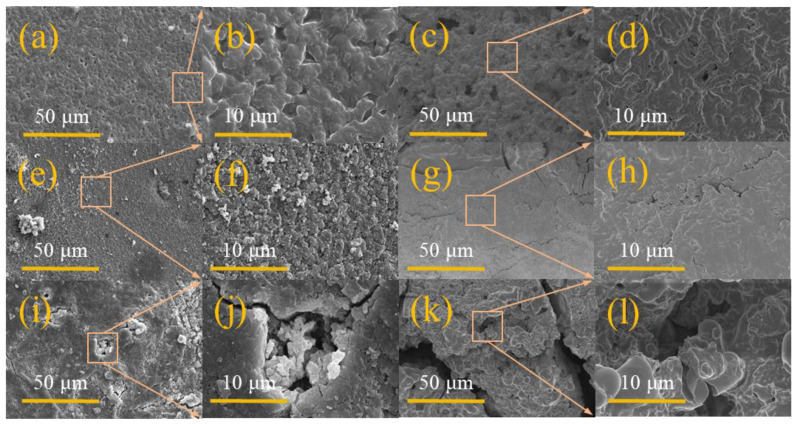
SEM images of GPEs before 100 cycles: surface (**a**,**b**); cross-section (**c**,**d**). SEM images of GPEs after 100 cycles in single-layer battery: surface (**e**,**f**); cross-section (**g**,**h**). SEM images of GPEs after 100 cycles in three-layer battery: surface (**i**,**j**); cross-section (**k**,**l**).

**Figure 4:**
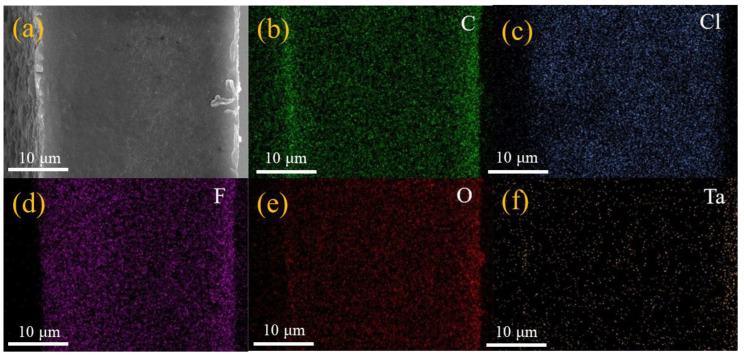
(**a**–**f**) Elemental distribution of C, Cl, F, O and Ta in GPEs.

Figure 3 shows the scanning electron microscopy (SEM) observation results of polymer electrolyte membranes under different states. In the initial samples before the experiment (Figure 3a–d), it can be observed that there are a large number of solid particles and uneven morphological characteristics on the surface of the sample. This situation may be related to the local aggregation of ceramic fillers (LLZTO) during the slurry drying process, and the crystallization precipitation formed during the curing stage can easily lead to an increase in surface roughness. It is worth noting that this structural feature will have an important impact on the mechanical properties of the electrolyte membrane. After 100 charge and discharge cycles, the electrolyte membrane (Figure 3e–h) in the single-layer battery showed significant structural deterioration. The surface image shows a large number of cracks and holes, especially the peeling phenomenon in the edge area of the film is more prominent. Judging from the cross-sectional image, the originally dense layered structure begins to appear loosely layered, which indicates that the electrolyte material may expand or shrink during long-term charging and discharging. This change in physical structure will directly weaken the material’s ability to inhibit lithium dendrites, thereby affecting the safety of the battery. Interestingly, in the test samples of multi-layer soft-pack batteries (Figure 3i–l), although the surface morphology looks relatively complete, abnormal grain growth still exists at the microscopic scale. The cross-sectional image shows obvious structural looseness, and the inter-layer bonding intensity in some areas is significantly reduced. This situation may be due to the uneven distribution of stresses generated in the multi-layer lamination process and the interface degradation caused by stress release during the cycle. In addition, the surface texture of the PTFE mold will affect the micromorphology of the electrolyte membrane through transfer, and the surface fluctuations formed by this transfer effect may change the contact state of the electrolyte with the electrode material. Through auxiliary analysis of element distribution map (Figure 4a–f), it can be found that the dispersion of LLZTO ceramic particles in the electrolyte membrane is relatively uniform. The design of this heterostructure has a dual mechanism: the smooth surface facing the positive electrode is rich in polymer components, which is conducive to reducing the interface impedance; while the rough surface facing the negative electrode is rich in ceramic fillers, which improves the contact performance with metal lithium through physical anchoring. However, experimental data also show that as the number of cycles increases, the synergy between the two interfaces gradually weakens, which may be one of the important reasons for the overall performance of the battery to decay. Figure 4a–f shows the dispersion of each element inside the GPEs material. Through the element distribution map, the existence and distribution characteristics of carbon, chlorine, fluorine, oxygen and tantalum elements in the material system can be visually observed, which provides basic spatial information support for subsequent analysis.

**Figure 5:**
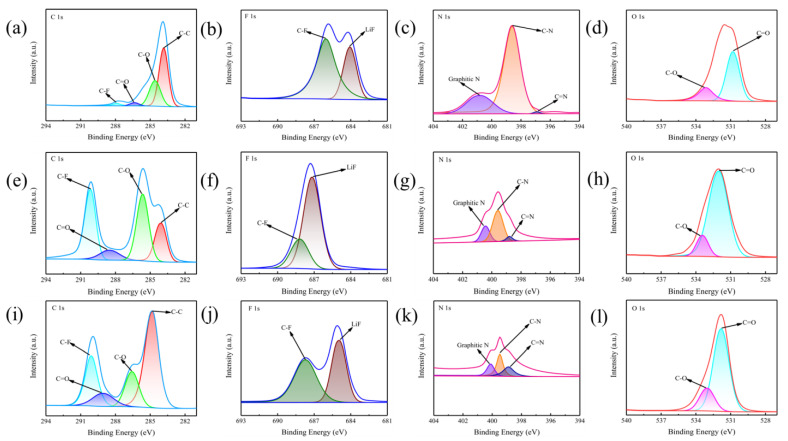
XPS spectra of GPEs under different states. (**a**–**d**), Before charge discharge cycle C1s, F1s, N1s and O1s; (**e**–**h**), After single-layer battery charging and discharging cycle C1s, F1s, N1s and O1s; (**i**–**l**), After three-layer battery charging and discharging cycle C1s, F1s, N1s and O1s.

The Figure 5 shows the C1s, F1s, N1s, O1s spectra of X-ray photoelectron spectroscopy (XPS) of GPEs membrane battery before charging and discharging cycle, after 100 charging and discharging cycles of single-layer GPEs membrane battery, and after 100 charging and discharging cycles of three-layer GPEs membrane battery. From the C1s energy spectrum (Figure 5a), it can be found that there are characteristic peaks in four locations: 284.78 eV, 286.20 eV, 288.68 eV and 290.52 eV. These characteristic peaks correspond to different types of carbon chemical bond structures, which belong to C-C single bond, C-O/C-N mixed bond, C=O double bond and C-F bond type, respectively. The O1s energy spectrum (Figure 5b) shows the main peak at 532.19 eV, and combined with the secondary peak at 533.41 eV, it fully proves that there are a large number of oxygen-containing groups in the material system, which is mainly related to the LLZTO ceramic filler added to the polymer matrix. Of particular concern are the two obvious peaks in the N1s energy spectrum (Figure 5c). After analysis, the former corresponds to the C=N double bond structure, while the latter comes from the C-N single bond system. In the F1s energy spectrum (Figure 5d), a strong signal at 686.8 eV indicates that the solid electrolyte interface (SEI) layer is rich in LiF components. By comparing the samples after different cycles (Figure 5f,g), it can be observed that with the complexity of the battery structure, the LiF content in the SEI layer showed a downward trend, while in contrast, the LiF content in the single-layer structure decreased more significantly. This change may affect the passivation effect of the lithium metal surface, because experimental data show that the presence of LiF has a positive effect on slowing down the formation of lithium dendrites. It inhibits the continuous reaction between lithium metal and electrolyte by forming a stable protective layer, which is of great significance to improving the cycle life of the battery.

Further observations found that after multiple charge and discharge cycles, not only did the LiF content change, but the peak intensity of other chemical bonds also had a certain deviation. For example, in the region corresponding to the C=O bond (Figure 5e), the cycled samples show a wider peak-shaped feature, which may imply that oxidative reconstruction of the surface layer of the material is performed. The peak difference in the N1s energy spectrum (Figure 5i,k) suggests that the chemical environment of nitrogen elements has undergone slight changes during the cycle, and this structural evolution may affect the stability of the ion transport channel. These phenomena together show that the electrode/electrolyte interface will undergo dynamic adjustment during repeated charging and discharge, and this interface reconstruction behavior is an important factor restricting the degradation of battery performance.

**Figure 6:**
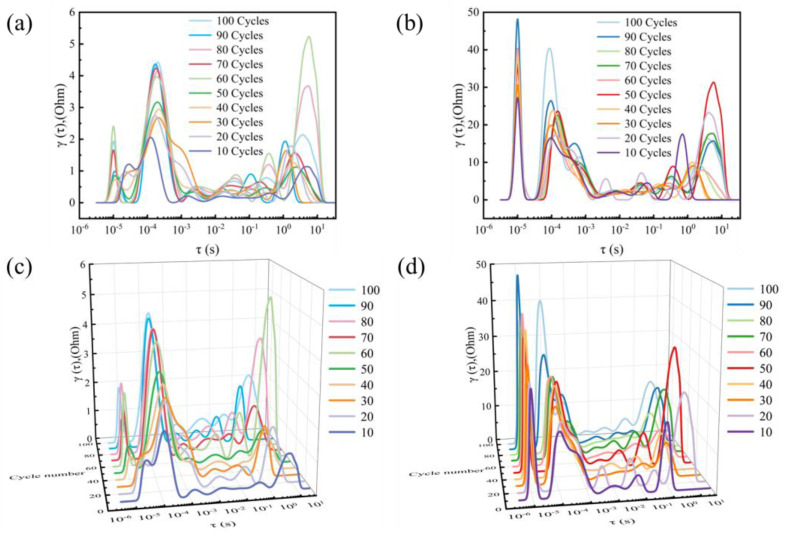
DRT analysis in EIS ((**a**,**c**) single-layer battery; (**b**,**d**) three-layer battery). The corresponding 3D evolution of the (**a**,**b**) is shown in the (**c**,**d**).

Electrochemical Impedance Spectroscopy (EIS) was conducted in situ for the full cell by performing EIS every 10 cycles during galvanostatic cycling. The measurement was done at the end of discharge after an hour of resting at the open circuit voltage. Deconvolution of the EIS data by DRT (distribution of relaxation times) transformation in the relaxation time space, as shown in Figure 6.

Quantitative assessment of the interfacial impedance evolution in batteries was performed via in-situ EIS. EIS enables real-time tracking of the evolution of ionic transport resistance at the interface under varying charge-discharge cycle numbers, providing critical evidence for understanding the rate and mechanisms of interfacial degradation. The three-layer pouch cell exhibited a significant increase in interfacial impedance after extended cycling, as shown in Figure 6b, where the ionic transport pathways became gradually obstructed with progressive cycling, leading to rapid capacity fade. In contrast, the single-layer pouch cell demonstrated a markedly slower growth in interfacial impedance after multiple cycles (Figure 6a). The arc-shaped evolution of the EIS spectra indicates more stable interfacial contact and sustained unimpeded ion transport.

**Figure 7:**
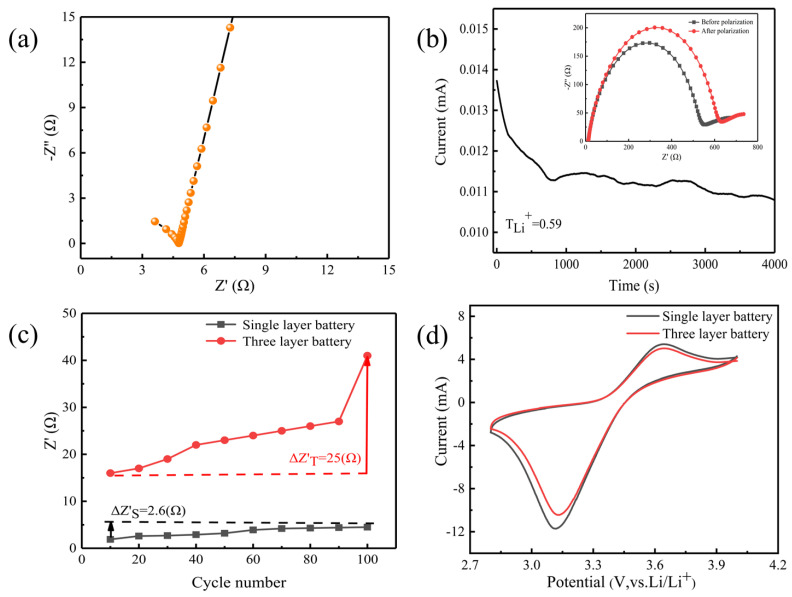
(**a**) Impedance diagram, (**b**) DC polarization and AC impedance curves of GPEs, (**c**) Impedance and charge discharge cycles, (**d**) CV curves.

Figure 7 system shows the multi-dimensional performance characterization of gel polymer electrolytes (GPEs). The impedance spectrum test results of Figure 7a show that the electrolyte membrane incorporated with LLZTO inorganic filler exhibits better conduction characteristics. From the perspective of microstructure, this may be because the addition of rigid ceramic particles destroys the regular arrangement of the polymer matrix, causing their crystallinity to significantly reduce. This disordered crystal structure can open up more migration paths for lithium ions, just like opening up multiple shortcuts for passage in dense forests. The impedance measured by EIS is 4.773 Ω and the ionic conductivity calculated by Formula (1) is 3.86 × 10^−4^ S·cm^−1^. This numerical comparison is more intuitive in the lateral comparison of Table 1.

Figure 7b displays the composite solid polarization curve and initial/steady-state impedance profile of the electrolyte membrane. Parameters: *I_0_* = 0.01492 mA, *I_SS_* = 0.01076 mA, *R_0_* = 552.123 Ω, *R_SS_* = 633.598 Ω. The lithium-ion transference number calculated using Equation (2) is 0.59. Furthermore, studies indicate that incorporating LLZTO ceramic fillers induces relaxation of polymer chains. Interactions between the inorganic fillers and polymer chains promote segmental motion and accelerate dynamic processes [51]. These interactions not only enhance ionic conductivity but also improve mechanical properties and interfacial stability of the electrolyte membrane. Battery impedance increases progressively during charge/discharge cycles. Comparative analysis reveals that the three-layer cell exhibits significantly greater impedance growth (Figure 7c).

Figure 7d presents cyclic voltammetry (CV) results for single-layer and three-layer GPEs within 2.8–4 V at a scan rate of 0.2 mV s^−1^. For the single-layer pouch cell, a distinct reduction peak at 3.15 V and a strong oxidation peak at 3.75 V correspond to lithium-ion insertion into and extraction from the LFP cathode, respectively. The substantially larger integrated redox peak area indicates higher specific capacity in the single-layer cell. Conversely, the three-layer pouch cell exhibits weak redox peaks, demonstrating inferior electrochemical performance.

**Figure 8:**
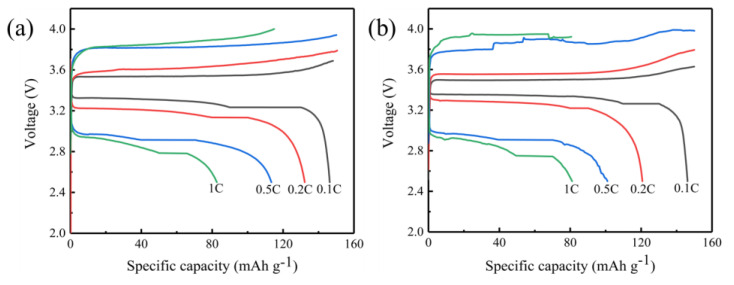
Charge-discharge curves. (**a**) single-layer soft-pack battery; (**b**) three-layer soft-pack battery.

The above differences are further verified by the comparison of charge and discharge curves in Figure 8. In the 2.5–4 V operating voltage range, batteries of both structures exhibit typical voltage platform characteristics during the initial charging and discharging process, which is consistent with the reversible deintercalation mechanism of lithium ions in lithium iron phosphate materials. However, under different current density conditions, the behavior of the two shows significant differences: when a small magnification of 0.1 C is used, the discharge capacity of the three-layer structure reaches 126.2 mAh g^−1^, which is higher than 106.3 mAh g^−1^ in the single-layer structure; however, after increasing the current to 0.5 C, the discharge capacity of the three-layer structure drops sharply to 71.1 mAh g^−1^, which is significantly lower than 83.4 mAh g^−1^ in the single-layer structure. This performance inversion phenomenon caused by changes in current density may be related to the differences in its internal ion transmission path.

**Figure 9:**
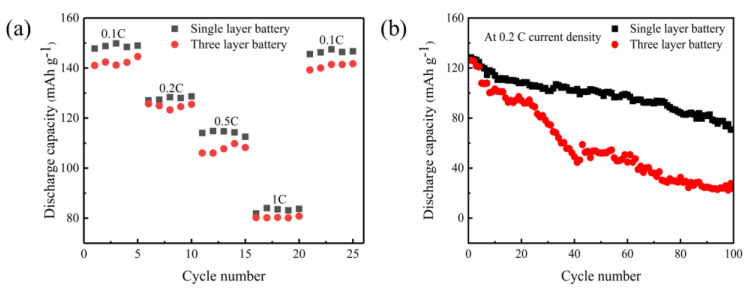
(**a**) Rate capability, (**b**) cycling performances.

The rate performance test results of Figure 9 show more fully this characteristic change trend. In the process of gradually increasing the discharge rate (0.1 C→0.2 C→0.5 C→1 C), although the single-layer soft-pack battery also exhibits capacity attenuation, its capacity retention rate at each stage is relatively stable. Especially when the 0.1 C ratio is adjusted back again, its capacity is basically restored to the initial value, showing good reversibility. In contrast, the capacity of the three-layer structure shows a cliff-like decline with the increase in magnification. Even under the conditions of high current of 1 C, its capacity is only comparable to that of a single-layer structure. This obvious performance decay may be related to the increase in charge transport impedance caused by its stacked structure, and the contact loss between multi-layer interfaces may be an important factor restricting its high current working ability.

It should be noted that there is a good mutual proof relationship between these electrochemical test results. The peak area difference in the voltammetric curve corresponds to the charge and discharge capacity data, and the performance deterioration trend of the three-layer structure in the magnification test also forms a logical chain with its smaller response peak intensity in the voltammetric test. These experimental phenomena together show that although multilayer structures may exhibit capacity advantages under certain specific conditions, their overall electrochemical stability and kinetic performance are still significantly inferior to those of single-layer structure design.

Figure 9b shows the cycle stability test results of assembled solid-state batteries under different structural conditions. The experiment selected a 0.2 C ratio for constant current charging and discharge, and compared the 100 cycles performance of single-layer and three-layer soft-pack batteries in the voltage range of 2.8 to 4.0 volts. Observing the data, it can be found that the initial discharge capacity of the two types of batteries is not much different. The starting capacity of the single-layer structure is 144.5 mAh g^−1^, and the three-layer structure is slightly higher than 151.1 mAh g^−1^. However, as the number of cycles increases, the capacity attenuation trend has significantly differentiated: by the 50th week, the single-layer battery still maintained a capacity of 100.3 mAh g^−1^ (about 69.4% of the initial value), while the three-layer structure has dropped sharply to 52.1 mAh g^−1^ (only 34.5% left); after continuous charging and discharging to 100 weeks, the single-layer battery capacity remained at 70.7 mAh g^−1^ (48.9% retention rate), but only 23.6 mAh g^−1^ (15.6% retention rate) remained at the three-layer battery. This shows that the monolayer configuration still has good electrochemical stability in the medium term, and the rapid decline of the three-layer structure indicates that there may be interface failure problems inside it.

**Figure 10:**
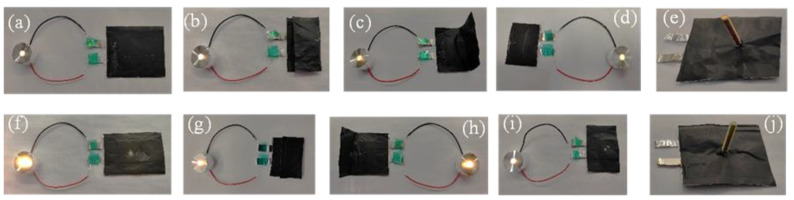
(**a**) Successfully working with the LED light before cutting or bending or needle puncture of the single-layer GPEs membrane soft pack battery, (**b**) After 180° bending, (**c**) After 90° bending, (**d**) After cutting half, (**e**) after needle puncture; (**f**) Successfully working with the LED light before cutting or bending or needle puncture of three-layer GPEs membrane soft pack battery, (**g**) After 180° bending, (**h**) After 90° bending, (**i**) After cutting half, (**j**) after needle puncture.

Figure 10 shows the LED lamp test photos of single-layer GPEs membrane soft pack battery and three-layer GPEs membrane soft pack battery under the conditions of cutting, bending at different angles, needling, etc. It can be seen from the figure that the LED can still light up after 90°, 180° bending, cutting and needling of both single-layer and three-layer GPEs film soft pack batteries, which indicates that the soft pack battery assembled based on GPEs has high safety.

## 3. Conclusions

In this paper, PVDF-HFP/PAN-LiClO_4_-LLZTO composite gel electrolyte was prepared through UV curing technology, and the performance differences between single-layer and three-layer soft-pack batteries were systematically compared. In the low-rate discharge test of 0.1 C, the three-layer structure battery exhibited a discharge specific capacity of 126.2 mAh g^−1^, which was significantly better than the 106.3 mAh g^−1^ of the single-layer structure. When the high magnification of 0.5 C was reached, the situation reversed, and the capacity of the three-layer structure dropped sharply to 71.1 mAh g^−1^, which was about 15% lower than the 83.4 mAh g^−1^ of the single-layer structure. This phenomenon shows that in application scenarios where rapid discharge is required, the design of a single-layer structure may have more advantages.

It is worth noting that under the medium magnification 0.2 C, the initial capacity of the two structures is basically at the same level. The single-layer battery is measured to be 144.5 mAh g^−1^, and the third-layer battery is 151.1 mAh g^−1^. However, as the number of cycles increases, the performance differentiation between the two begins to appear. After 50 charge and discharge cycles, the single-layer battery can still maintain a capacity of 100.3 mAh g^−1^, while the capacity of the three-layer battery has dropped significantly to 52.1 mAh g^−1^. In terms of capacity retention, the single-layer structure has a stability of 69.4%, but the three-layer structure has a residual capacity of only 34.5%. This rapid attenuation may be due to interface contact problems caused by multi-layer stacking.

In view of the existing experimental results, subsequent research needs to focus on solving the interfacial impedance problem in multi-layer structures, try polymer-ceramic composite materials with different ratios, and perhaps find a combination solution with better conductivity. At the same time, the battery’s high and low temperature adaptability should be systematically evaluated, after all, the actual use environment is much more complicated than that in the laboratory. It should also be noted that the current test is mainly carried out at the laboratory level. To advance to the industrial application stage, more comprehensive performance verification is also required in the complete battery system. In particular, in-depth exploration is needed in terms of electrode material matching and packaging process optimization.

## 4. Materials and Methods

### 4.1. Preparation of PVDF-HFP/PAN-LiClO_4_-LLZTO Gel Polymer Electrolyte

The preparation of GPEs adopts a UV curing process, and the specific operation process is shown in Figure 10. First, 12 g of N, N-dimethylformamide (DMF, Aladdin, Shanghai, China, 99.8%) was weighed into a beaker, and a magnetic stirrer was activated at 45 °C and 300 rpm. Subsequently, 1.4 g of PVDF-HFP (Mn = 600,000, Zhanyang, Dongguan, China) and 0.6 g of PAN (Zhanyang, Dongguan, China) were dissolved/dispersed in the DMF, and the stirring speed was increased to 500 rpm. After thorough mixing, 0.6 g of LiClO_4_ (Aladdin, Shanghai, China, 99%) was added while maintaining the temperature at 45 °C, followed by 5 min of stirring. Next, 0.2 g of LLZTO (Kcrystal, Shenzhen, China, 99%) was added and stirred for 5 h. After this period, 0.8 g of ETPTA and 0.5 g of 425 were introduced and stirred for 5 min. Finally, 3 drops of HMPP were added and stirred for an additional 5 min.

**Figure 11:**
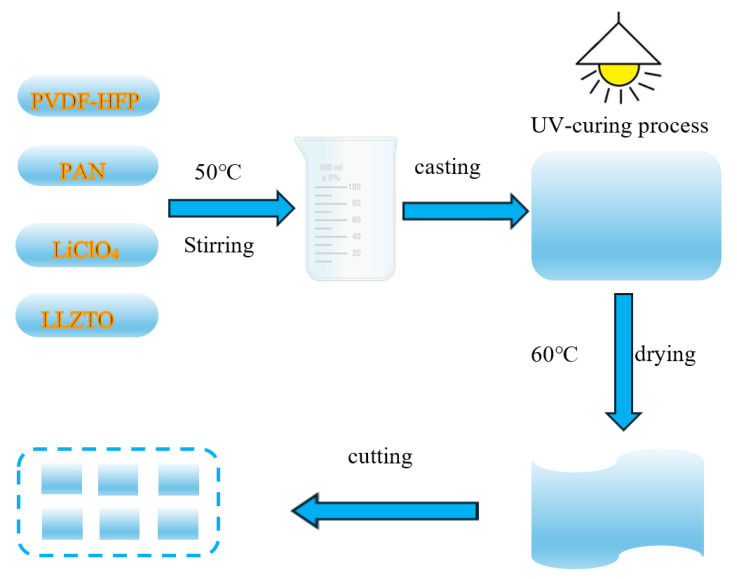
Schematic pattern of the preparation process of GPEs.

After cooling for 5 min, the mixed slurry was transferred to a polytetrafluoroethylene (PTFE) mold for static casting. Following coating onto a PTFE plate, the gel electrolyte membrane was cured using a UV curing machine at a speed of 0.01 m/min for 10 s under controlled conditions (30 °C, wavelength 365 nm). After vacuum drying at 60 °C for 40 min, the resulting PVDF-HFP/PAN-LiClO_4_-LLZTO GPEs could be easily peeled off. Finally, the electrolyte membrane was cut into rectangular pieces measuring 48 mm long and 28 mm wide, and stored in an argon-filled glove box (<0.01 ppm H_2_O and O_2_). The thickness of the PVDF-HFP/PAN-LiClO_4_-LLZTO membrane prepared by solution casting was measured as 37 μm using a micrometer screw gauge. The preparation flowchart of GPEs is shown in Figure 11.

### 4.2. Material Characterization

Phase composition analysis employed XRD (DX-2700, Manufacturer: Dandong Haoyuan Instrument Co., Dandong, China; Cu-Kα radiation). Surface morphology and chemistry were characterized via SEM (Phenom Pharos G2, Manufacturer: Shanghai FEI Ltd. Shanghai, China) integrated with XPS spectroscopy. Raman spectra acquisition utilized an ATR8000 confocal microscope (Manufacturer: Fujian FocusTek, Fuzhou, China). TGA measurements proceeded on a Netzsch F3 Tarsus system (Bavaria, Germany) under argon purge, implementing a dynamic temperature program (30→800 °C, β = 10 °C/min).

### 4.3. Electrode Preparation

N-methylpyrrolidone (3 drops) was introduced into a borosilicate beaker. Polyvinylidene fluoride (0.2 g) was dissolved under magnetic stirring (300 rpm, RT). Separately, a blended powder of LiFePO_4_ (1.6 g) and carbon black (0.2 g) was ground in an agate mortar (40 min). The powder mixture was dispersed into the PVDF/NMP solution and stirred (4 h, RT). The resultant slurry was coated via doctor blade (50 μm gap) onto current collectors, followed by vacuum drying (60 °C, 36 h). Dried electrodes were cut to 44 mm × 24 mm with 12 mm × 8 mm tabs and stored under argon atmosphere.

### 4.4. Battery Assembly

**Figure 12:**
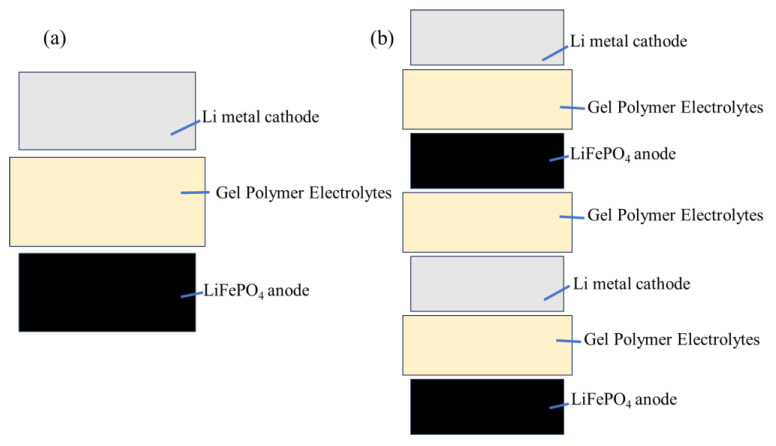
The schematic diagrams of the assembled. (**a**) single-layer batter, (**b**) three-layer battery.

The soft-pack battery is assembled by stacking. First, the negative electrode copper-lithium composite strip is cut into a 44 mm long, 24 mm wide, 12 mm long, and 8 mm wide electrode, which is matched with the cut positive electrode. The first layer is the negative electrode, followed by the cut gel electrolyte membrane of 48 mm long and 28 mm wide, and then the positive electrode of the same size as the negative electrode. The multi-layer soft-pack battery is stacked in this way. After the stacking is completed, tape is attached to the four sides of the soft-pack battery, and then 0.8 MPa and 1.0 MPa are applied on the sheet press (Manufacturer: ZJ-D33-YP15 Beijing Jingke Zhichuang Technology Development Co., Ltd. Beijing, China) respectively for single-layer and three-layer soft-pack batteries, so that the interface between the soft-pack battery electrode sheet and the gel electrolyte membrane is better contacted. After the sheeting is completed, the pole ear is welded on the ultrasonic spot welding machine (Manufacturer: Shenzhen Kejing Equipment Co., Ltd., Shenzhen, China, MSK-800). The size of the pole ear is 0.1 * 8 * 46 mm. The positive pole ear is an aluminum pole ear, and the negative pole ear is a nickel pole ear, which is welded on the positive current collector aluminum foil and the negative current collector copper foil respectively. Then cut the aluminum plastic film with a length of 65 mm and a width of 55 mm, set the parameters on the vacuum pre-sealing machine (Manufacturer: Xinwei Equipment Co., Ltd., Shenzhen, China,), the temperature is 150 °C, the time is 3 s, and one side of the aluminum plastic film is encapsulated. Then put the battery batteries with the pole ear welded into the aluminum plastic film and encapsulate it on the top. Then inject LiPF_6_ electrolyte into the soft pack battery, and finally set the vacuum degree to −93 kPa in the vacuum pre-sealing machine to encapsulate the last side. The design capacity of the single-layer soft pack battery is 20 mAh, and the design capacity of the three-layer soft pack battery is 60 mAh. The assembled soft-pack battery was aged at 55 °C for 8 h, then left at room temperature for 16 h, then charged at 0.05 C for 2 h, then charged at 0.1 C for 3 h, and finally aged at 55 °C for 24 h. After aging, the soft-pack battery was vented and repackaged for testing. Figure 12a,b show the schematic diagrams of the assembled single-layer and three-layer pouch batteries, respectively.

### 4.5. Electrochemical Properties Test

EIS was performed on an electrochemical workstation (DH 7000, Donghua, Nanjing, China) using a symmetric button battery assembled with a stainless steel SS/GPEs/SS structure. The frequency range of the EIS test was 10^6^ Hz to 0.1 Hz with an amplitude of 10 mV.

The electrochemical stability window of the GPE was evaluated by linear sweep voltammetry (LSV) using a semi-symmetric SS/GPEs/Li cell within a voltage range of 2.5 V to 6.0 V at a scan rate of 5 mV/s. The lithium-ion transference number (*T_Li_*^+^) of the GPE was quantified via Bruce-Vincent method using symmetric Li||GPEs||Li cells, combining chronoamperometry (DC polarization at 10 mV) with EIS (100 kHz–0.1 Hz) using the same electrochemical workstation.

The calculation formula of ionic conductivity σ is as follows: [34]

(1) $$\sigma =\frac{L}{RS}$$

where:

*σ* is the ionic conductivity (S/cm).

*L* denotes the thickness of the membrane (cm).

*R* represents the local minimum resistance (Ω) in the full impedance spectrum (typically the bulk resistance *R_b_* from EIS Nyquist plot).

*S* indicates the contact area between the membrane and stainless steel (SS) electrodes (cm^2^).

The Lithium ion transference number is an important parameter for evaluating the ion migration rate [52].

The calculation formula of lithium ion transfer numbers as follows: [53,54]

(2) $$T_{{Li}^{+}}=\frac{I_{SS}(∆V-I_{0}R_{0})}{I_{0}(∆V-I_{SS}R_{SS})}$$

where:

$T_{{Li}^{+}}$ denotes the lithium-ion transference number in the electrolyte.

*I_0_* and *I_SS_* represent the current values before and after DC polarization stabilization (mA).

*R_0_* and *R_SS_* are the resistances before and after DC polarization (Ω).

Δ*V* is the voltage of 10 mV applied across the battery.

## Figures and Tables

**Table 1:** Contrastive Evaluation of Emerging and Established Gel Polymer Electrolytes.

GPEs	Ionic Conductivity (×10^−4^ S/cm)	Ref.
PAP/PEP	4.27 (RT)	[42]
CEPs	0.81(25 °C)	[43]
CSE (15 wt.% LLZTO)	0.55 (30 °C)	[44]
PPPL-10	4 (25 °C)	[45]
PEO/PVDF/NaNO3	0.93 (25 °C)	[46]
PEO-PVDF-LITFSI	2.46 (25 °C)	[47]
SPEs (PLiZ)	1.19 (25 °C)	[48]
PPLG (5 wt.% graphene)	3.39 (25 °C)	[49]
SSCEs	2.91 (25 °C)	[50]
GPEs	3.86 (RT)	This work

## Data Availability

The data are contained within the article.
